# Beyond Beauty: Onobotulinumtoxin A (BOTOX®) and the Management of Migraine Headaches

**DOI:** 10.5812/aapm.6286

**Published:** 2012-07-10

**Authors:** Devra Becker, Bardia Amirlak

**Affiliations:** 1 Department of Plastic and Reconstructive Surgery, Case Western University, Cleveland, USA; 2 Department of Plastic and Reconstructive Surgery, University of Texas Southwestern Medical Center, Dallas, USA

**Keywords:** Onabotulinumtoxin A, Headache, Migraine Disorders, Trigger points, Neuralgia

## Abstract

Based on the conducted anatomic studies at our institutions as well as clinical experience with migraine surgery, we have refined our onobotulinumtoxin A (BOTOX®) injection techniques. Pain management physicians are in unique position to be able to not only treat migraine patient, but also to be able to collaborate with neurologists and peripheral nerve surgeons in identifying the migraine trigger sites prior to surgical deactivation. The constellation of migraine symptoms that aid in identifying the migraine trigger sites, the potential pathophysiology of each trigger site, the effective methods of botulinumtoxin and nerve block injection for diagnostic and treatment purposes, as well as the pitfalls and potential complications, will be addressed and discussed in this paper.

## 1. Introduction

Migraine headaches (MH) represent a significant cause of morbidity, not only for the prevalence of the disorder, but for the economic burden and the decrease in quality of life. There has been increasing interest and several large-scale studies on the use of onobotulinumtoxin A (BOTOX®) for the treatment of MH (1-8). In 2010 the Food and Drug Administration (FDA) approved onobotulinumtoxin A for the use in prevention of headaches in adults with chronic migraine.

Surgical deactivation of migraine trigger sites as well as preoperative botulinum toxin injection has been proven effective for the treatment of severe MH (9-13). Based on the “trigger point” hypothesis of migraine pathogenesis, plastic surgeons introduced the use of botulinumtoxin and nerve blocks as a diagnostic adjunct and predictor of responsiveness for migraine surgery. There is now increasing literature documenting the efficacy of onobotulinumtoxin A for short and long-term treatment of variety of pain craniofacial pain states as well other than MH (14-17).

There is a distinction between the work that has been done on onobotulinumtoxin A in the prevention of chronic migraines (5-8) and the work that has been done by plastic surgeons to confirm suspected trigger sites (10). To understand this, it is first important to understand the types of MH, the outcome measures of studies on onobotulinumtoxin A in the treatment of MH, and the trigger point hypothesis and anatomic considerations used by plastic surgeons in management of MH.

## 2. Diagnosis and Classification of Headaches

Because headaches in themselves are common, and many types of headaches can be confused with MH, it is important to establish a diagnosis of MH prior to initiating treatment, either medical or surgical. Diagnostic criteria for migraine headache are outlined by the International Headache Society (IHS). The headaches are typically unilateral, pulsating, and are typically aggravated by physical activity. In addition, MH are associated with nausea and/or vomiting, photophobia, or phonophobia. MH are further subdivided into those with aura (in which the aura develops over five to twenty minutes and last less than one hour) and without aura. MH are further subdivided into episodic and chronic migraines. Although typically distinguished by the number of headache days per month (episodic migraines are defined by fewer than fifteen headache-days per month, and chronic migraines are defined by more than fifteen headache days per month over a three-month period), population studies have shown that patients with chronic migraines have significantly more comorbidities, including depression, anxiety, chronic pain, respiratory disorders, cardiac risk factors, and were less likely to have full-time employment. Buse and coauthors note that these findings are consistent with a more nuanced and complex understanding of the differences between chronic migraines and episodic migraines (18).

Onobotulinumtoxin A is approved by the FDA for use in prevention of headaches in chronic, but not episodic migraines. The Phase III Research Evaluating Migraine Prophylaxis Therapy I (PREEMPT I) trial was designed as a phase 3 study, with a 24-week double-blind, placebo controlled arm followed by an open label arm. The treatment arm consisted of thirty one fixed-site, fixed dose injections in seven anatomic areas, although studies investigators were able to administer 40 u as “follow-the-pain” strategy, in addition to the designed 155 u. The study noted that while there was no difference between onobotulinumtoxin A and placebo for the primary endpoint (headache episodes), reductions in the onobotulinumtoxin A group were found for other measures, including headache and migraine days, cumulative hours of headache, and frequency of moderate/severe headache days (6). The pooled data from the PREEMPT trials suggested that prophylactic treatment with onobotulinumtoxin A improved the health-related quality of life (HRQoL) in chronic migraines across a number of measures, thus reducing the disease burden (7, 8). Thus while onobotulinumtoxin A use did not eliminate migraines, it appeared effective in converting chronic migraine to episodic migraine (19), although whether this has resulted in changes of comorbidities such as depression, anxiety, or overall work-related disability has not been established. Studies of onobotulinumtoxin A use in episodic migraines, however, have failed to show an improvement over placebo (20). In this meta-analysis of eight randomized, double-blind, placebo-controlled trials, however the injection techniques in the sites studied differed from our focused anatomical injection techniques.

Prior to any intervention, it is critical that a patient be evaluated by a neurologist to confirm the diagnosis of migraine headache, and to identify possible secondary causes of headache, such as medication overuse headache.

## 3. Trigger Sites

Migraine pathogenesis is known to be a complex, multifactorial process that involves neuronal hyper excitability during the interictal phase, cortical spreading depression as the basis of aura (21, 22), dysfunctional periaqueductal gray matter (23), the development of cutaneous allodynia during a migraine attack, clinical evidence for the sequential recruitment of spinal and supraspinal nociceptive neurons in migraine (24), and trigeminal nerve activation at a peripheral and central origin that accounts for the headache and progressive central sensitization (25). The trigeminal nerve irritation releases of Substance P, calcitonin gene-related peptide, and neurokinin, a localized meningitis and the progression of pain follows (26-30).

The trigger point hypothesis attempts to explain what the initial trigger of trigeminal nerve irritation is. It takes into account that many patients describe the onset of migraine as occurring in a specific area, and that each migraine may have different characteristics depending on the site of onset. Thus, irritation of peripheral nerves by muscular contraction, or by contact points that induce inflammation, is what ‘triggers’ MH.

This theory was first proposed by neurologists and later validated after making the clinical observation of alleviation of migraine symptoms in those patients who underwent endoscopic excision of the corrugator muscles and surgical dissection of the supraorbital and supratrochlear nerves during forehead lifts (1, 9, 31, 32). This lead to further clinical and anatomical research on various trigger sites, based largely on the work of Guyuron et al. (10). The surgical procedures base their protocols on the trigger point hypothesis of migraine pathogenesis (33, 34).

Studies of onobotulinumtoxin A by neurologists for prevention in chronic migraine have been designed with injection of many anatomic sites (20). Based on the “trigger point” hypothesis of migraine pathogenesis, Plastic Surgeons introduced the use of onobotulinumtoxin and nerve blocks as a diagnostic adjunct and predictor of responsiveness for migraine surgery (10, 35, 36). Thus, the use of onobotulinumtoxin A by Plastic Surgeons has traditionally been for a different purpose than MH prevention; it is used as a way to identify potential sites of decompression. The surgical approach is to try to identify specific trigger areas to tailor pain management, and to identify sites of potential surgical decompression. In this approach, all potential sites of peripheral triggers can be deactivated as a preventative strategy as well. This specific tailored anatomical use in our clinical experience has resulted in positive response in both episodic and chronic migraines.

Trigger sites are identified by the anatomic site in which patients report the migraines begin, and not the final location of the migraine which can be present in more than one site. In order to be able to identify this accurately we obtain a thorough history from the patient, and provide the patient with a Migraine Diary Chart (MDC) to fill daily for at least four weeks prior to labeling the offending trigger sites. Since patients often report the general location of the migraines and sometimes do not pay attention to the trigger origin site, this diary is of paramount importance. The injection must be done in the trigger site where the pain originates and not necessarily where the pain ends. In general, there are four potential trigger sites that have relevance to the surgeon today: frontal (Site I), temporal (Site II), occipital (Site IV), and rhinogenic (Site III).

Frontal triggers begin in the central and lateral forehead, and the pain has a central and cephalad radiation. At times they are associated with supraorbital and supratrochlear tenderness. The pain is generally described as being “above the eye radiating from outside to in”. In some cases, corrugator supercilii muscle (CSM) hypertrophy may also be present. Patients in general have a more prominent vertical rhytid associated with the more active corrugator muscle, which could correspond to the laterality of the migraines. Direct pressure over the supraorbital and supratrochlear areas may increase or decrease the pain.

Temporal triggers are associated with lateral temporal and posterior auricular radiation of pain. Patients may also note temporal muscle ‘tightness.’ Temporomandibular joint pathology should be excluded as the trigger for migraines.

Occipital triggers starts with pain in the occipital region and radiate anteriorly. Patients may also complain of retroauricular pain. The specific area where the migraine starts is usually localized based on the anatomical location of the greater occipital nerve and can be identified by direct digital pressure over the location where the nerve pierces the semispinalis capitis muscle. Atmospheric pressure change may also trigger occipital pain, presumably through a change in greater occipital artery vascular tone, which lies in close proximity to the greater occipital nerve (37).

In septal triggers, patients often report an association with the weather and pressure change usually starting during morning hours. They may note rhinitis, halitosis, dental pain, and hyposmia or anosmia. Septal triggers should also be considered if patients have continued complaints despite injection on onobotulinumtoxin A in other trigger sites. The pain is generally described as being “behind the eye from coming from inside to out”. Patient also commonly complain of breathing problems. Treatment with oxymetazoline nasal spray may temporarily halt the progression of pain from this site and serve as a diagnostic adjunct. A computed tomography scan can confirm intranasal pathology, such as contact between the septum and turbinates, and conchae bullosae that can contribute to MH, and often can be addressed surgically (38).

These trigger sites, then, determine the surgical approach. Although surgical intervention for migraine surgery has been described before (39-41), it has recently been elaborated using endoscopic and other techniques that are associated with less dissection and shorter incisions (13). These surgical interventions include resection of the zygomaticotemporal branch of the trigeminal nerve through an endoscopic technique, resection of the corrugator muscle to relieve pressure on the supraorbital and supratrochlear nerves, release of the greater occipital and related nerves, and intranasal surgery for rhinogenic triggers of migraine. Surgical studies over the past twelve years, primarily published in the Plastic Surgery literature, have supported the efficacy of these surgical procedures (1, 3, 11, 12).

## 4. Use of Onobotulinumtoxin A

While migraine descriptions by patients provide information for trigger site selection, the injection itself is performed based on the anatomy of the trigger sites. Recent anatomical studies have provided increased knowledge about the musculature and nervous anatomy of the trigger sites (2, 42, 43).

## 5. Frontal Migraines and Technique of Injection

The supraorbital and supratrochlear nerves are thought to be compressed by the corrugator supercilii muscle, but any of the other glabellar muscles (depressor supercilii, and procerus) may be involved. Janis *et al*. (44) noted four separate branching patterns of the supratrochlear nerve through the CSM. The CSM itself is a triangularlyshaped muscle whose origin is the supraciliary arch and inserts laterally into the dermis.

**Figure 1:**
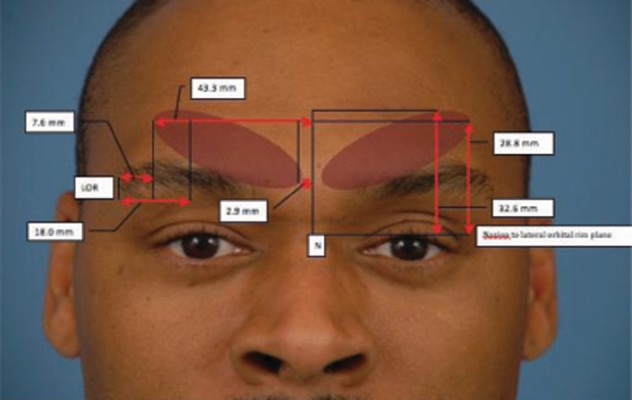
Comprehensive Corrugator Supercilii Muscle Topographic Anatomy and Measured Points of the Corrugator Supercilii Muscle in Relation to the Palpable Bony Anatomy. The vertical midline connecting the nasion (N) and anterior nasal spine is used as the reference landmark from midline. Lateral Orbital Rim (LOR) is used as the lateral reference landmark

**Figure 2:**
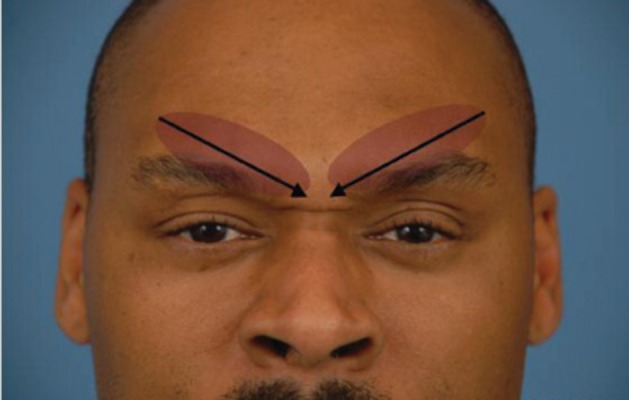
Anatomical Injection Patient is asked to frown to better delineate the corrugator supercilii muscle. Needle is inserted from lateral extent of the muscle to medial from superficial to deep.

**Figure 3:**
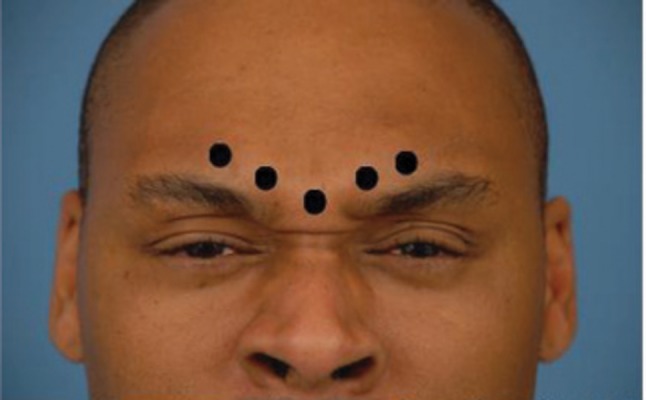
Five Point Injection Patient is asked to frown to better delineate the corrugator supercilii muscle.

For injection, we use a 1 cc tuberculin syringe with 0.01 gradations and a long 30 g needle. The CSM is marked during dynamic motion, and areas of hypertrophy are delineated. The muscle itself begins approximately 3mm lateral to middle, and extends 43 mm laterally. It extends 33 mm superiorly from the pupil (*Figure 1*). To minimize the volume and possible caudal leakage of the drug causing inadvertent complication of eyelid ptosis, this solution is more concentrated than the ones used for the temporal and occipital regions. A total of 12.5U in 0.25 cc of NS is injected into each corrugator, deeper medially to reflect the anatomy, and more superficial laterally. The needle is inserted from superficial lateral border of the corrugator to the medial deep surface (*Figure 2*). The injection can be done either while drawing the needle back or advancing it, but we prefer injecting as advancing, which provides more control of the needle. Alternatively, a five-point injection technique can be used (*Figure 3*). Skin cooling with ice packs before and after injection increases the patient comfort. After injection, gauze is place on the injection site with light pressure. Massage of the site is avoided, as it may cause diffusion which may lead to eyelid ptosis. We do not inject onobotulinumtoxin A into the frontalis muscle at this time, as there is no evidence for an anatomic basis for compression.

Anatomical compression sites for the supratrochlear and supraorbital nerve, other than glabellar muscles, can exist. Such sites include a cascade of supratrochlear and supraorbital vessels, and at anatomic variants of the supraorbital notch (such as a foramen, or a facial band across the notch) (45, 46). These structural variations can contribute to compression of the nerves and may be a cause of onobotulinumtoxin A failure. In such instances, relieving the nerve irritation through either endoscopic nerve decompressions or transpalpebral nerve decompression may help with MH (47).

## 6. Temporal Migraines and Technique of Injection

**Figure 4:**
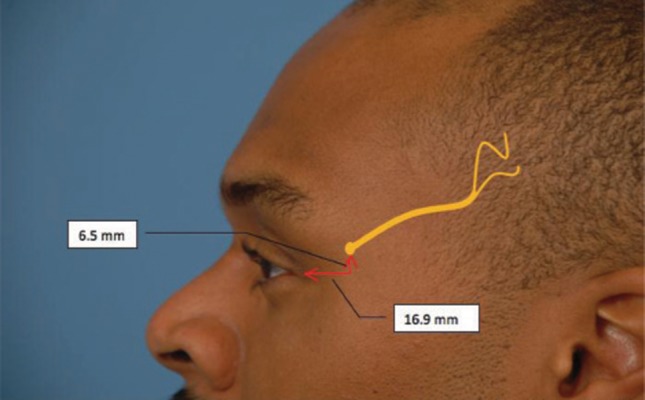
A Hollow Area Surrounds the Point of Emergence of the Main Branch of the Zygomaticotemporal Branch of the Trigeminal Nerve Which Can Be Located by Palpation. (Average Distance 16.9 mm Posterolateral and 6.5 mm Cephalad to the Lateral Palpebral Commissure)

**Figure 5:**
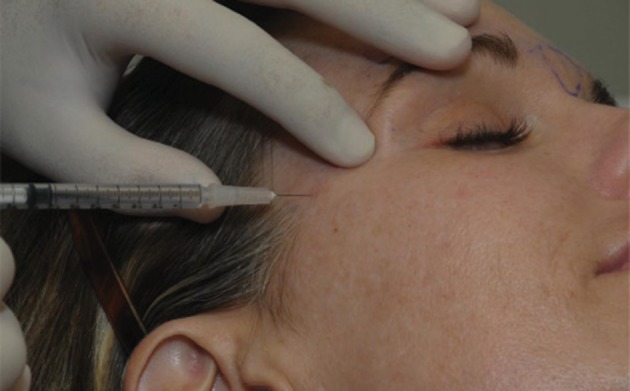
Method of Botulinum Toxin A Injection in Temporal Area Needle is inserted 2 cm lateral to the emergence site of the zygomaticotemporal branch of the trigeminal nerve (Marked by index or long finger) and is advanced through the deep temporal fascia

Anatomic studies have shown that the zygomaticofrontal branch of the trigeminal nerve arises 16.9 mm posterolateral to the commissure and 6.5 mm cephalad to the lateral commissure (*Figure 4*) (2). The technique of injection into the temporal muscle is the same as the glabellar muscles. We identify the location of the nerve based on the anatomic studies above, by palpating the emergence of the nerve in the temporal fossa, inject 2cm posterior to that point (*Figure 5*). The needle should pierce the temporalis fascia, and 12.5 U in 0.5 cc NS should be injected with a long 30 g needle for a radius of 1.5 cm. Occasionally, patient may experience temporary atrophy of the muscles due to the effects of onobotulinumtoxin, which can appear as “hour glassing” on frontal view.

## 7. Occipital Triggers and Technique of Injection

**Figure 6:**
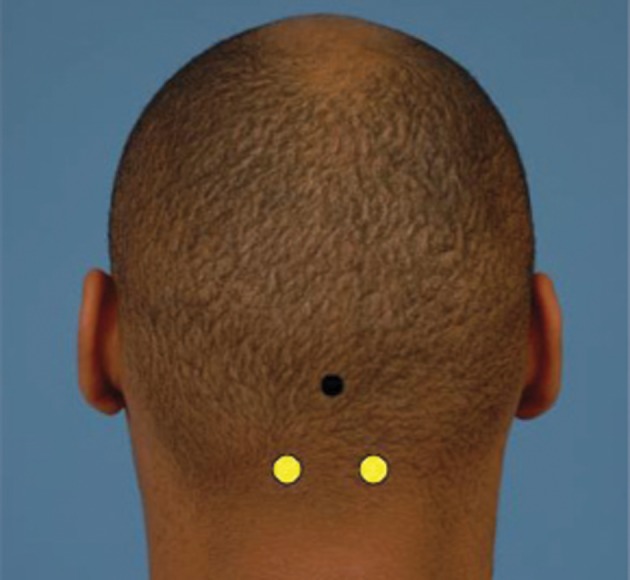
Sites of Emergence of the Greater Occipital Nerves (White Dot) is Located 1.5 cm Laterally and 3 cm Inferiorly to the Occipital Protuberance (Black Dot)

The greater occipital nerve is located approximately 1.5 cm from the midline and 3 cm caudal to the occipital protuberance. The nerve pierced the semispinalis capitis muscle in 98.5% of cases, and was asymmetric in approximately half of specimens in one study (*Figure 6*) (4, 48).

We use 25 U of onobotulinumtoxin A in 0.5 cc of NS injected per side using the landmarks shown in *Figure 6*. For this the patient is placed in a prone position with hands above the head and the drug is injected deep into the semispinalis capitis and splenius capitis muscles. Nonresponders may have other anatomic causes; one study noted that trapezius fascia and the occipital artery may also play a role in nerve irritation (37, 43).

Onobotulinumtoxin A can also be used in cases of occipital neuralgia (17). Occipital neuralgia presents with unilateral or bilateral radiating pain characterized by piercing or throbbing in the occipital region, and behind the ears. Nerve block may identify occipital neuralgia more readily in acute setting. The response to onobotulinumtoxin A is good but often delayed in these cases.

## 8. Algorithm and Sequence of Injections

Appropriately selected patients may then undergo aonobotulinumtoxin A trial injection in various trigger sites to determine whether they could benefit from surgical intervention. If intervention with onobotulinumtoxin A yields a sustained elimination of migraines or significant improvement, defined as at least 50% reduction from baseline intensity and/or frequency for at least four consecutive weeks, surgical management should be considered for each known trigger.

The summary of the sequence is as follows:

1. Migraine Diary for four weeks (documents the location where the migraine starts, frequency, duration and intensity of each trigger site).
2. Onobotulinumtoxin A for primary and secondary triggers (Sites I, II, and lV only)
3. CT scan for site III
4. Diary for 6 weeks
5. If no additional triggers, proceed with surgery
6. If additional triggers, proceed with injections
7. Diary for 6 weeks
8. Decision for surgery; Selected patients will undergo surgery for deactivation of frontal, temporal, retro-bulbar (behind the eyes), or occipital MH either at one setting or as serial surgeries depending on the dominance of a specific trigger site.

This time course may not be feasible for out-of-town patients; however, reliable detection of migraine triggers can still be achieved by using local nerve blocks, (during the onset of migraine only) a single-stage injection of onobotulinumtoxin A into the predominate trigger site, and/or computed tomographic confirmation of intranasal pathology. The above step-wise injection can be an alternative for treatment purposes for reasons of cost saving.

New potential trigger sites have come out of anatomic studies (42, 49). While addressing these are not currently part of common clinical practice, they may be in the future. At times, dormant trigger sites are masked by stronger ones and deactivation of the stronger sites by onobotulinumtoxin A may manifest symptoms at the new site. Once can elect to ignore the lesser aggressive site after deactivation of a main trigger if the pain is relatively tolerable or well controlled with medication. An algorithmic and conservative approach to injections will not only lessen the potential side effects but will result in cost saving.

## 9. Complications

Complications of Onobotulinumtoxin A injections to sites I, II and IV although rare, could include eyelid ptosis, hourglass deformity of the temples, and early fatigue during neck extension and diplopia. Hourglass deformity may improve over time, but can be corrected with artificial fillers or fat injections. Apraclonidine 0.5% eye drops may help with eyelid ptosis. Apraclonidine is an 2-adrenergic agonist, which causes Müller muscles to contract quickly elevating the upper eyelid. By minimizing the amount of onobotulinumtoxin A injected to the necessary amount by correct identification of the trigger sites and other maneuvers described above one can minimize these complications.

## 10. Summary

We use onoboutlinumtoxin as an aid in identifying migraine trigger points. Anatomic variations must be understood so that the onobotulinumtoxin A can be effectively injected at trigger point sites. Knowledge of anatomy for the pain management physicians will provide a more effective, efficient and cost saving way to deliver the Onobotulinumtoxin A for treatment of migraines and craniofacial pain syndromes. This can be done both as a temporary treatment strategy or as a diagnostic tool for trigger sites that can later be decompressed by a migraine surgeon.
